# Ratiometric fluorescence imaging of cell surface pH by poly(ethylene glycol)-phospholipid conjugated with fluorescein isothiocyanate

**DOI:** 10.1038/s41598-017-17459-y

**Published:** 2017-12-13

**Authors:** Ryuichi Ohgaki, Yuji Teramura, Daichi Hayashi, Lili Quan, Suguru Okuda, Shushi Nagamori, Madoka Takai, Yoshikatsu Kanai

**Affiliations:** 1 Department of Bio-system Pharmacology, Graduate School of Medicine, Osaka University, 2-2 Yamadaoka, Suita, Osaka 565-0871 Japan; 2 Department of Bioengineering, The University of Tokyo, 7-3-1 Hongo, Bunkyo-ku, Tokyo, 113-8656 Japan; 3 Department of Immunology, Genetics and Pathology (IGP), Rudbeck Laboratory C5:3, Uppsala University, SE-751 85 Uppsala, Sweden

## Abstract

Various physiological and pathological processes are accompanied with the alteration of pH at extracellular juxtamembrane region. Accordingly, the methods to analyze the cell surface pH have been demanded in biological and medical sciences. In this study, we have established a novel methodology for cell surface pH imaging using poly(ethylene glycol)-phospholipid (PEG-lipid) as a core structure of ratiometric fluorescent probes. PEG-lipid is a synthetic amphiphilic polymer originally developed for the cell surface modification in transplantation therapy. Via its hydrophobic alkyl chains of the phospholipid moiety, PEG-lipid is, when applied extracellularly, spontaneously inserted into the plasma membrane and retained at the surface of the cells. We have demonstrated that the PEG-lipid conjugated with fluorescein isothiocyanate (FITC-PEG-lipid) can be used as a sensitive and reversible cell-surface-anchored pH probe between weakly alkaline and acidic pH with an excellent spatiotemporal resolution. The remarkably simple procedure for cell-surface labeling with FITC-PEG-lipid would also be advantageous when considering its application to high-throughput *in vitro* assay. This study further indicates that various probes useful for the investigation of juxtamembrane environments could also be developed by using PEG-lipid as the core structure for bio-membrane anchoring.

## Introduction

The interphase between plasma membrane and extracellular fluid has been attracting considerable attentions of researchers, and various analytical techniques are under development^1^. The pH of extracellular juxtamembrane region, especially acidic pH of the cellular surface, is critical in various physiological and pathological processes. The surface of intestinal epithelia is covered with an acidic unstirred water layer^2,3^ which affects the intestinal absorption of drugs and xenobiotics as well as the luminal-membrane nutrient transport^4,5^. Osteoclasts actively pump out proton and acidify their surroundings for bone resorption^6^. The exterior of solid tumor is known to be acidic because the excretion of metabolic acids is increased due to the elevated aerobic glycolysis. Such acidification of tumor surroundings is proposed to be associated with tumor metastasis^7^ and chemotherapy resistance^8,9^. The measurement and visualization of the extracellular juxtamembrane pH, therefore, holds significant biological and medical implications.

Fluorescence-based imaging techniques in general have great advantages because of their wide application potential, high sensitivity, and excellent spatiotemporal resolution. The ratiometirc approach is especially powerful because it allows the measurements independent of the probe concentration. Recently, several fluorescent pH probes anchored at the cell surface have been reported for ratiometric analyses. They include a genetically-encoded pair of pH-dependent and -independent fluorescent proteins^10^, a FRET-pair fluorophores conjugated with pH-dependent single stranded DNA (ssDNA) structural-switch^11^, a pair of pH-sensitive and -insensitive fluorophores conjugated with lipid-ssDNA^12^ and a ratiometric pH-sensitive fluorophore bound with a pH low insertion peptide (pHLIP)^13^. However, probes for the cell surface pH imaging with a desired spatiotemporal resolution and a simple cell-surface labeling have not been available.

In the transplantation therapy, the cell surface engineering using natural and synthetic polymers has been proposed to circumvent the recipient’s immune response and improve the graft survival^14,15^. An amphiphilic polymer, poly(ethylene glycol)-conjugated phospholipid (PEG-lipid), was originally designed for the encapsulation and the immune-isolation of pancreatic islet cells in the islet transplantation therapy of the type I diabetes^16^. It has also been applied to the surface modification of liposome-based synthetic erythrocyte to improve the biocompatibility and prolong the circulation time *in vivo* ^17^. When the PEG-lipid is added to the culture media of cells and islets, the alkyl chains of phospholipid moiety are rapidly and spontaneously inserted into the lipid bilayer of the plasma membrane via the hydrophobic interaction. It is stably retained at the plasma membrane for several hours without significantly affecting the cell viability^18–20^. Therefore, PEG-lipid has ideal characteristics for the use as the core structure of probes that anchor appropriate indicator moieties at the plasma membrane to measure the pH of extracellular juxtamembrane region. PEG-lipid conjugated with fluorescein isothiocyanate (FITC-PEG-lipid) was synthesized previously to visualize the localization of PEG-lipid in the cells^16^. A widely used fluorophore FITC exhibits a pH-indicative property, i.e. the ratiometric readout of its fluorescence takes unique values according to pH^21^. In this study, we have explored a novel application of FITC-PEG-lipid to the use of a ratiometric fluorescent probe to visualize the cell surface pH.

## Results

### pH-dependent fluorescent properties of FITC-PEG-lipid suspended in solution

**Figure 1:**
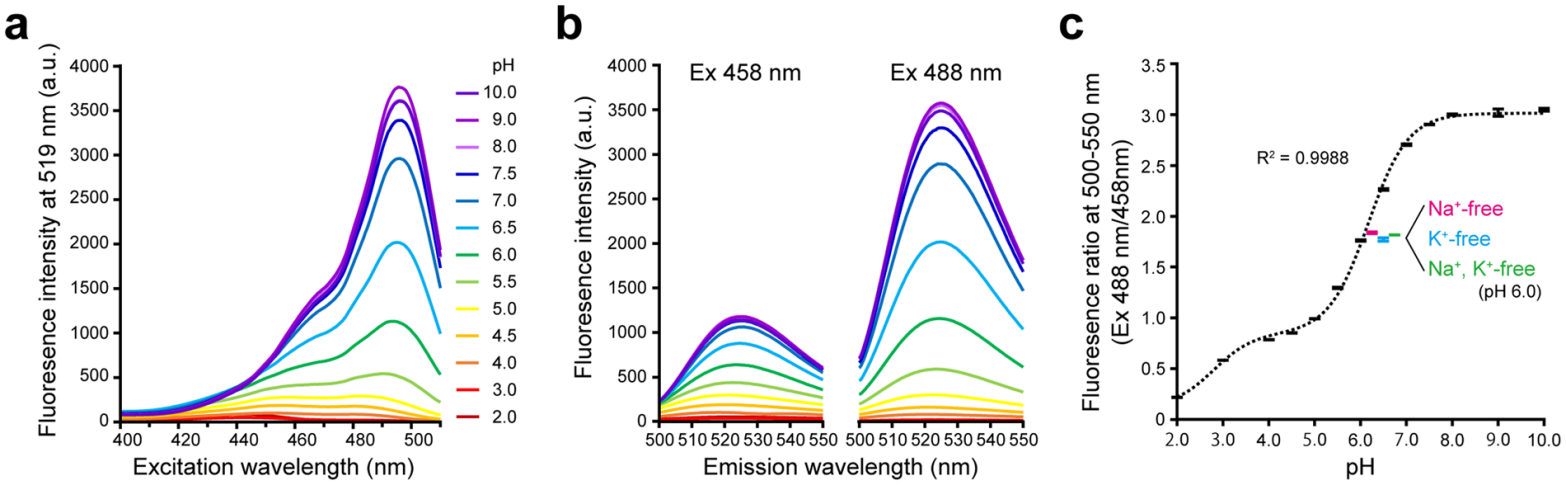
pH-dependent fluorescent property of FITC-PEG-lipid suspended in solution at various pH values. (**a**) Excitation fluorescence spectrum of FITC-PEG-lipid measured with emission at 519 nm. (**b**) Emission fluorescence spectra of FITC-PEG-lipid when excited at 458 nm (left) and 488 nm (right). The measurements of the spectra in (**a**) and (**b**) were performed in the buffered solutions with various pH (pH 2.0–10.0) as indicated with different colors. (**c**) Plot of the fluorescence ratio (Ex 488 nm/458 nm) against the pH of the buffered solutions (Black error bars). The fluorescence ratio was calculated from the integrated fluorescence intensity at 500–550 nm when excited at 458 nm and 488 nm. The dashed curve was obtained by fitting to a biphasic two-site competition ligand binding curve in non-linear regression analysis. Data of measurements in Na^+^-free, K^+^-free, and Na^+^, K^+^-free solutions at pH 6.0 are shown with colored error bars as indicated. Error bars represent SD (n = 4).

We first investigated whether FITC sustains its pH-dependent fluorescent properties even after conjugation with PEG-lipid. FITC-PEG-lipid was suspended in a series of buffered solutions of different pH (pH 2.0–10.0). Then, the excitation spectra (400–510 nm) were recorded by spectrofluorometer at the detection wavelength of 519 nm (Fig. 1a). The fluorescence from FITC-PEG-lipid exhibited a main peak at 495 nm and a shoulder peak at 465 nm, and showed a clear pH-dependence. To validate our ratiometric confocal laser scanning microscopy using Argon laser lines (458 nm and 488 nm) with a detection path of 500–550 nm, the emission spectra were recorded in the identical setting (Fig. 1b). At both excitation wavelengths, the fluorescence intensity of FITC-PEG-lipid was higher at alkaline-neutral pH and lower at acidic pH. The obtained ratio of integrated fluorescence (500–550 nm) was plotted against pH in Fig. 1c, illustrating the pH-indicative property of FITC conjugated with PEG-lipid. The plot was fitted to a biphasic two-site competition ligand binding curve. The results implied that two distinct protonation sites are dominantly affecting the fluorescence ratio of FITC-PEG-lipid. The inflection points were at pH 2.79 and 6.17, which are close to the previously reported pKa values of FITC for the cation-neutral form (pKa = 2.19) and monoanion-dianion form (pKa = 6.33), respectively^22^. The fluorescence ratio took unique values in a range of pH 2.0–7.5. However, due to the low fluoresence intensity at acidic pH and the less dynamic response around pH 4.5, the optimal detection range of FITC-PEG-lipid was determined to be between weakly alkaline and acidic pH. The fluorescence ratio was only negligibly affected when Na^+^ and/or K^+^ were omitted from the buffered solutions at pH 6.0, demonstrating the selectivity of the probe for proton over these physiologically abundant monovalent cations (Fig. 1c).

### Cell surface labeling with FITC-PEG-lipid

**Figure 2:**
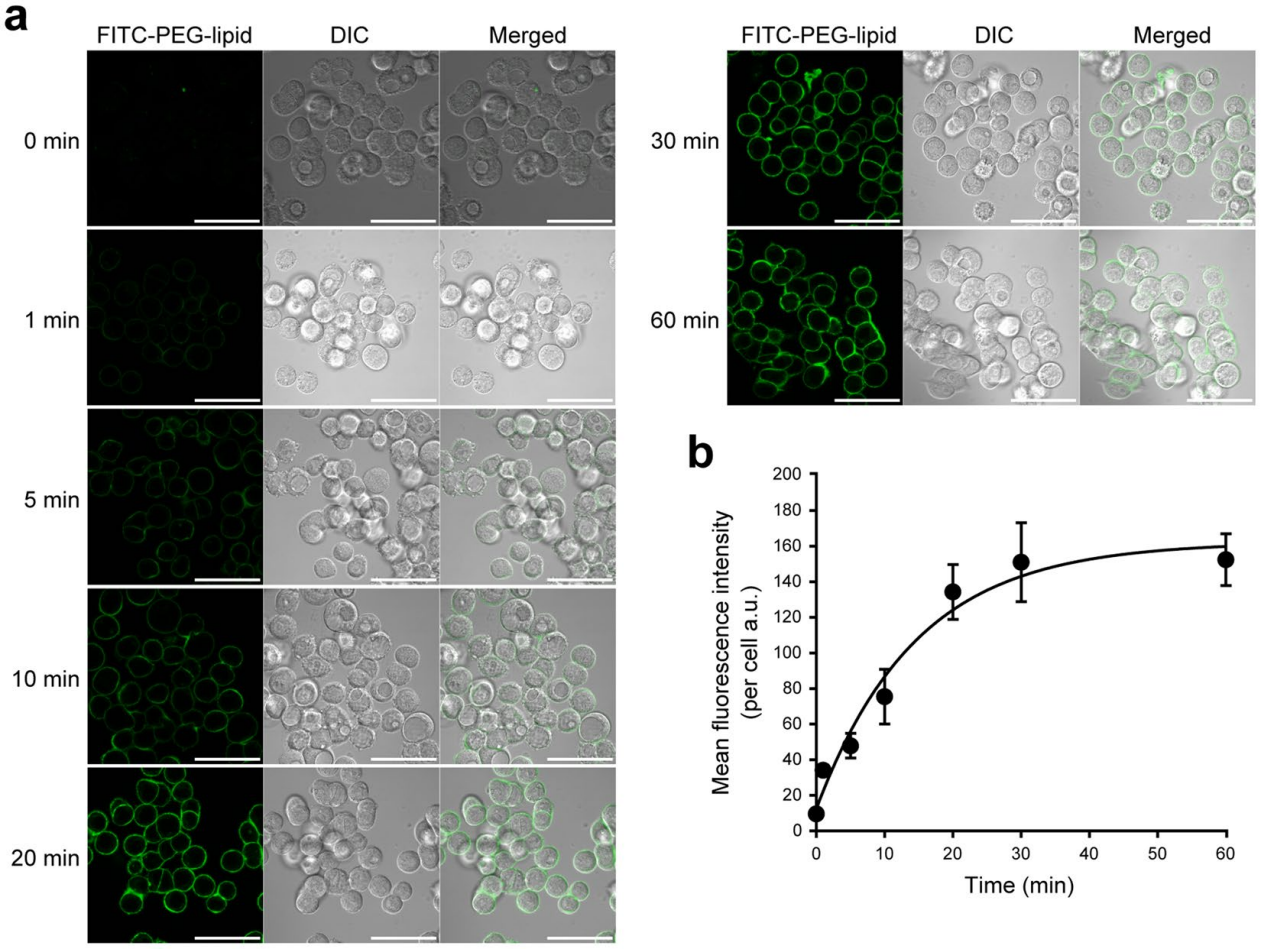
Time course of the cell surface labeling with FITC-PEG-lipid. Human gastric signet-ring-cell carcinoma KATO III cells were incubated with FITC-PEG-lipid for the indicated periods (0–60 min) at room temperature and subjected to confocal laser scanning microscopy with excitation at 488 nm and detection at 500–550 nm. (**a**) Representative images of confocal laser scanning microscopy of KATOIII cells labeled with FITC-PEG-lipid. At each time point, the fluorescent images of FITC-PEG-lipid (green, left panels), DIC images (gray scale, middle panels) and their merged images (right panels) are shown. Scale bars: 50 μm. (**b**) Plot of the mean fluorescence intensity of FITC-PEG-lipid per cell against the incubation time. The curve was obtained by fitting to a three-parameter single exponential model in non-linear regression analysis. Equation (1): Mean fluorescence intencity per cell = y_0_ + a × (1 − exp(−b × Time), where y_0_ = 12.5323, a = 149.7483, b = 0.6684. Error bars represent SD (n = 3–5).

To optimize the condition for imaging analysis, we next examined a time course of the cell surface labeling with FITC-PEG-lipid (Fig. 2). Human gastric signet-ring-cell carcinoma KATO III cells were incubated with FITC-PEG-lipid for 0, 1, 5, 10, 20, 30 and 60 min and subjected to confocal laser scanning microscopy. The fluorescence of FITC-PEG-lipid was exclusively detected at the plasma membrane. Significant internalization of fluorescence into the cytoplasm was not observed at least up to 60 min. This is well consistent with the cell surface retention property of FITC-PEG-lipid previously reported using other cell lines. From the images obtained at each incubation time, the mean fluorescence intensity per cell was calculated and plotted against the incubation time (Fig. 2b). The fluorescence intensity of FITC-PEG-lipid at the cell surface of KATO III cells reached the plateau at 30 min. Therefore, the incubation time for FITC-PEG-lipid labeling was fixed to 30 min in all the following experiments. It was confirmed that incubation with FITC-PEG-lipid does not significantly affect the viability of KATO III cells in this condition (Supplementary Fig. S1).

**Figure 3:**
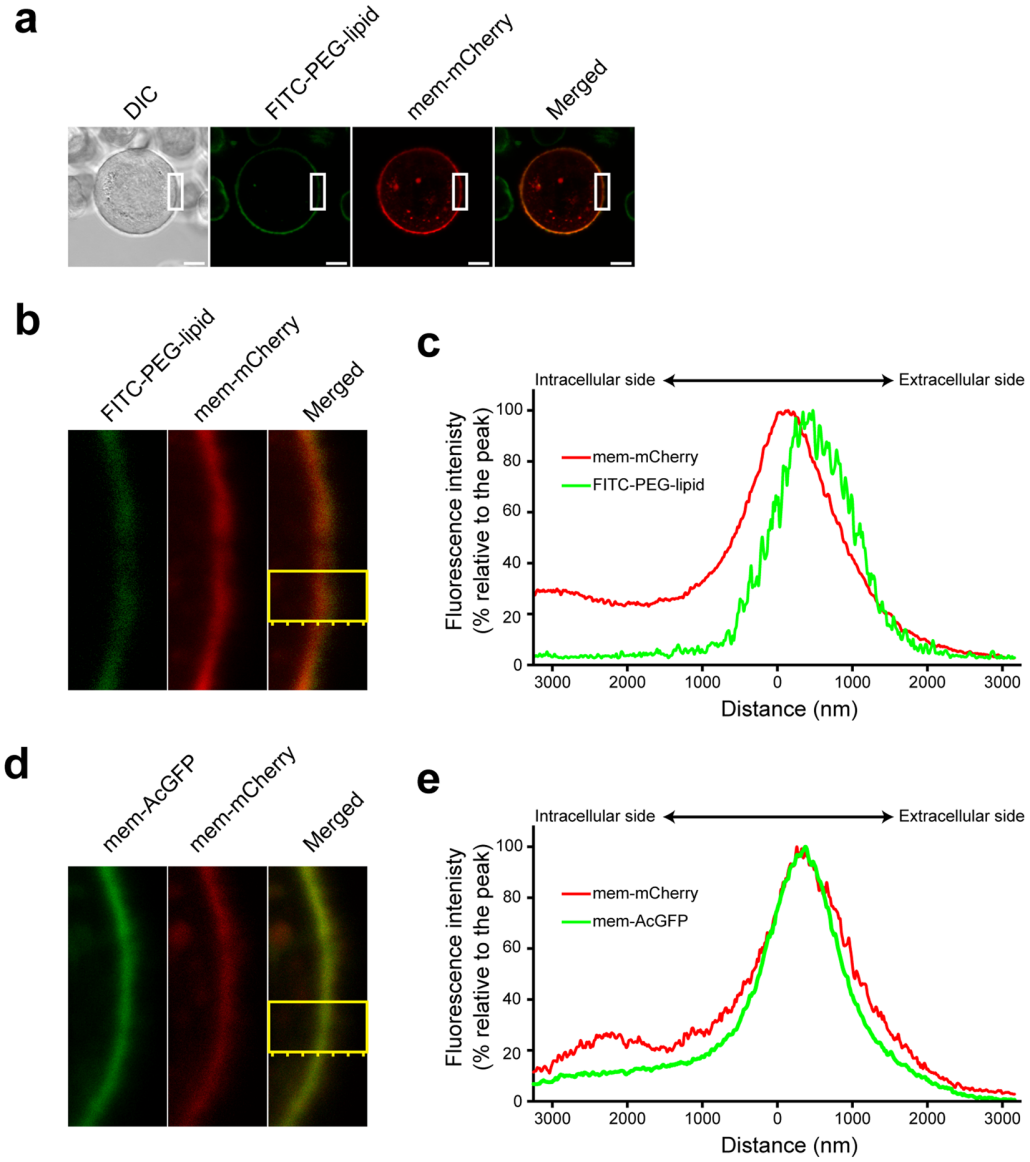
Localization of FITC-PEG-lipid at the cell surface. (**a**–**c**) KATOIII cells expressing mem-mCherry were labeled with FITC-PEG-lipid and subjected to confocal laser scanning microscopy. (**a**) Low magnification images. DIC image (gray scale), fluorescent images of FITC-PEG-lipid (green) and of mem-mCherry (red), and their merged image are shown. Scale bars: 10 μm. (**b**) Enlarged fluorescent images of the area indicated with white squares in (**a**). (**c**) Fluorescence peak detection for FITC-PEG-lipid and mem-mCherry across the plasma membrane in the area indicated with a yellow square in (**b**). (**d**,**e**) KATOIII cells co-expressing mem-mCherry and mem-AcGFP were subjected to confocal laser scanning microscopy. (**d**) High magnification fluorescent images of mem-AcGFP (green) and of mem-mCherry (red), and their merged image are shown. (**e**) Fluorescence peak detection for mem-AcGFP and mem-mCherry across the plasma membrane in the area indicated with a yellow square in (**d**). The normalized fluorescence intensities (% relative to the peak) of FITC-PEG-lipid, mem-AcGFP, and mem-mCherry were plotted against the distance from the center of the analyzed area.

To further confirm the retention of FITC-PEG-lipid at exterior cellular surface, mem-mCherry (a fusion protein of the *N*-terminal peptide of Lyn kinase and red fluorescent mCherry) was expressed in KATO III cells. The *N*-terminal sequence of Lyn kinase accepts fatty acylations which are necessary and sufficient to anchor the protein at the inner leaflet of the plasma membrane^23^. As shown in Fig. 3a, both FITC-PEG-lipid and mem-mCherry were localized at the plasma membrane. Observation with a higher magnification (Fig. 3b) and the peak detection across the plasma membrane (Fig. 3c) revealed that the fluorescent signals from FITC-PEG-lipid and mem-mCherry are mostly overlapping, but the fluorescence peak of FITC-PEG-lipid is slightly shifted toward exterior compared with that of mem-mCherry. When mem-AcGFP (a fusion protein of the *N*-terminal peptide of Lyn kinase and green fluorescent AcGFP) was co-expressed with mem-mCherry, such a shift in the fluorescence peak was not observed (Fig. 3d,e). These results further supported that FITC-PEG-lipid is retained at the extracellular surface of KATO III cells.

### Measurement of the cell surface pH using FITC-PEG-lipid

**Figure 4:**
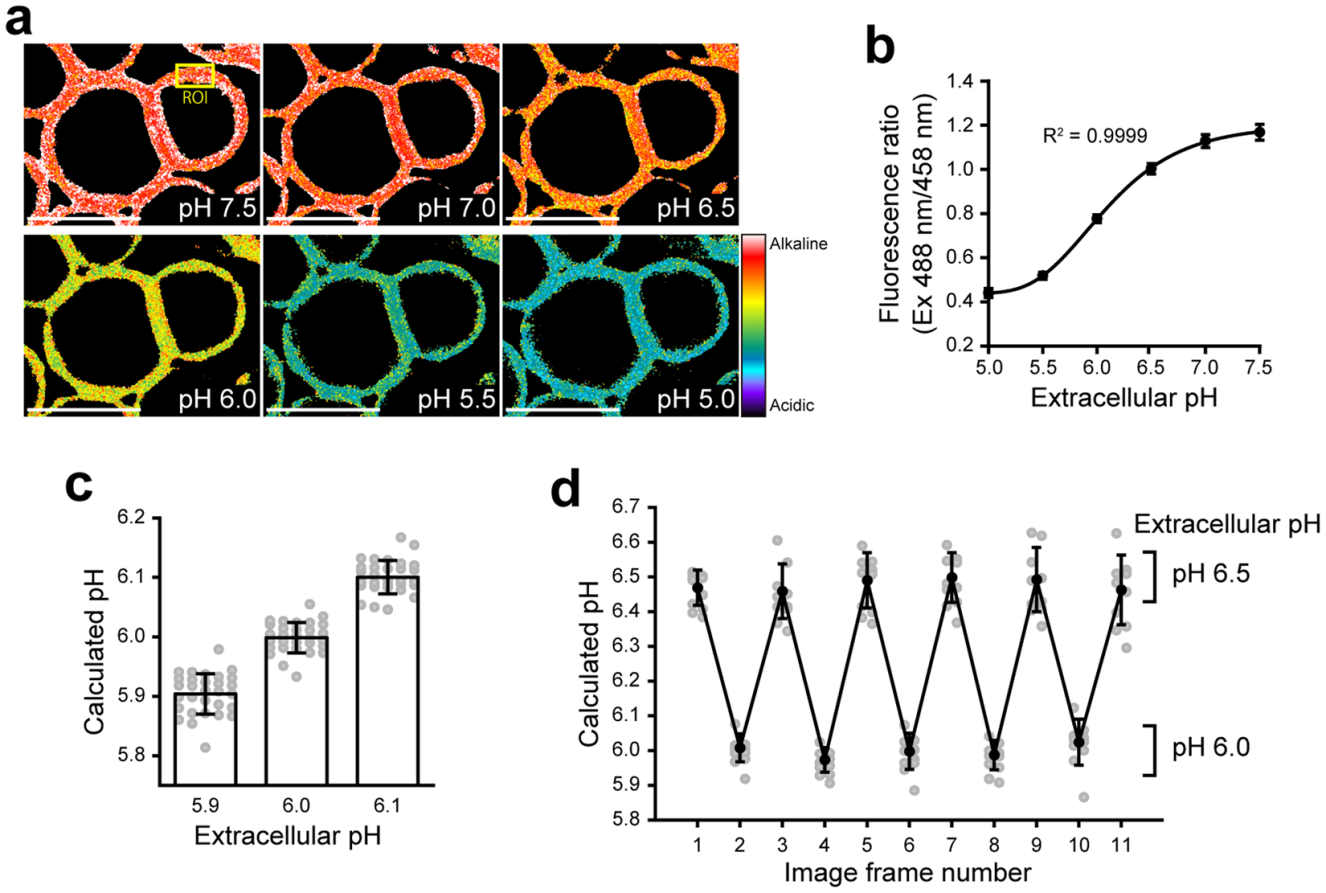
Ratiometric analysis of the cell surface pH using FITC-PEG-lipid. KATOIII cells labeled with FITC-PEG-lipids were subjected to confocal laser scanning microscopy in various external pH. Images were acquired at the excitation wavelengths of 458 nm and 488 nm, with the fluorescence detection range of 500–550 nm. (**a**) Pseudocolored fluorescence ratio images (Ex 488 nm/458 nm) acquired during the step-wise external pH shift from pH 7.5 to 5.0. Scale bars: 20 μm. An example of “ROI” (region of interest) used for the quantification of local cell surface pH in (**b**–**d**) is indicated with a yellow square. (**b**) Plot of the fluorescence ratio in ROIs against the pH of external buffers. The curve was obtained by fitting to a five-parameter sigmoidal model in non-linear regression analysis. Data shown are mean ± SD (n = 12). (**c**) Measurement of the local cell surface pH in external buffers of pH 5.9, 6.0 and 6.1. The local cell surface pH in ROIs was calculated from the fluorescence ratio using the Equation (2) (see text). Data shown are mean ± SD (n = 30). The plot of each ROI (n = 30) is shown with gray circles. (**d**) Reversible response of FITC-PEG-lipid to the repetitive external pH alteration. Images were acquired by changing the external pH between pH 6.5 and 6.0 repetitively. Data shown are mean ± SD (n = 10). The plot of each ROI (n = 10) is shown with gray circles.

To test whether FITC-PEG-lipid can be applied to the quantitative imaging of the cell surface pH, KATO III cells labeled with FITC-PEG-lipid were subjected to the ratiometric confocal laser scanning microscopy. During the analysis, the external pH was shifted step-wisely from pH 7.5 to 5.0 by manually exchanging the external buffered solutions. Measurements were not performed at more acidic pH, due to the weak fluorescence intensity that is near the lower detection limit of the confocal laser scanning microscopy under the current settings. Images were obtained in each solution at the excitation wavelengths of 458 nm and 488 nm, with the fluorescence detection range of 500–550 nm. As expected, the fluorescence ratio (488 nm/456 nm) was unique to the external pH and, therefore, represented with different colors in the pseudocolored images (Fig. 4a). For the quantitative analysis, regions of interest (ROIs) were set on the plasma membrane (as exemplified in Fig. 4a, ROI), in which the fluorescence ratio was calculated. The plotting of fluorescence ratio against pH of the external solutions demonstrated that the ratiometric readout of the FITC-PEG-lipid fluorescence well fit to the following five parameter sigmoidal equation by non-linear regression analysis (Fig. 4b). The parameters were set as follows: x_0_ = 4.3950, y_0_ = 0.4378, a = 0.7590, b = 0.4773 and c = 23.8004.

2 $${\rm{p}}{\rm{H}}={{\rm{x}}}_{0}-\,{\rm{l}}{\rm{n}}[{({\rm{a}}/{\rm{F}}{\rm{l}}{\rm{u}}{\rm{o}}{\rm{r}}{\rm{e}}{\rm{s}}{\rm{c}}{\rm{n}}{\rm{c}}{\rm{e}}{\rm{r}}{\rm{a}}{\rm{t}}{\rm{i}}{\rm{o}}-{{\rm{y}}}_{0})}^{1/{\rm{c}}}-1]\times {\rm{b}}$$

**Figure 5:**
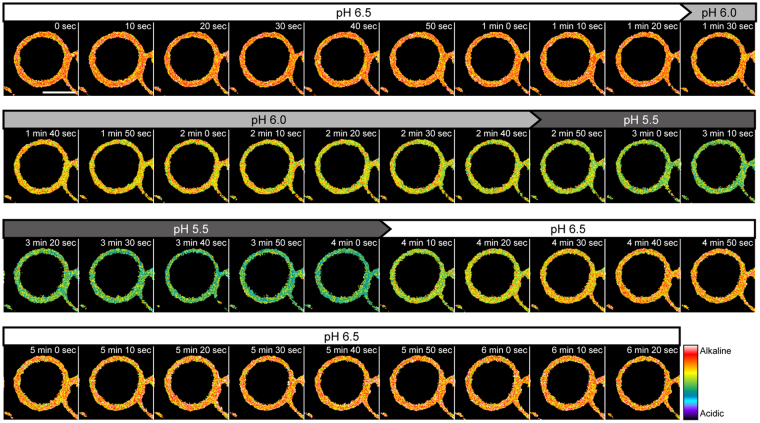
Time-lapse imaging analysis of the cell surface pH using FITC-PEG-lipid. KATOIII cells labeled with FITC-PEG-lipids were subjected to a time-lapse imaging analysis. Images were acquired at the excitation wavelengths of 458 nm and 488 nm, with the fluorescence detection range of 500–550 nm. The external solutions were seamlessly switched by using a stage-top perfusion system in the following order: pH 6.5, 6.0, 5.5 and recovery to pH 6.5. The pH values of the perfused external solutions are shown above the psudocolored fluorescence ratio images. Interval: 10 sec. The total duration: 6 min 20 sec (39 frames). Scale bar: 10 μm.

The measurement was accurate enough to distinguish the difference of 0.1 pH unit for the external solutions at pH 5.9, 6.0 and 6.1 (Fig. 4c). The local cell surface pH calculated from the fluorescence ratio using the Equation (1) was consistent to the actual external pH (pH 5.903 ± 0.034, pH 5.999 ± 0.025 and pH 6.100 ± 0.028; Mean ± SD, n = 30). Moreover, at least five times of repetitive changes of the extracellular pH between 6.5 and 6.0 did not significantly affect the calculated pH (Fig. 4d). Such a reversible response of FITC-PEG-lipid indicated its potential application for tracking the continuous pH fluctuation. In a time-lapse analysis in which the extracellular pH was seamlessly changed by a stage-top perfusion system, the fluorescence ratio was rapidly and reversibly shifted in response to the extracellular pH between 6.5 and 5.5 (Fig. 5 and Supplementary Video S1).

## Discussion

Extracellular acidification is observed in various scenarios in our body under physiological and pathological conditions^2–6^. Therefore, the development of functional probes for the visualization of cell surface pH holds significant biological and medical implications. In this study, we have demonstrated a novel application of FITC-PEG-lipid as a ratiometric fluorescent pH probe for external cell surface pH imaging. Using cultured cells, the cell surface pH was successfully monitored with a good spatiotemporal resolution in both static and time-lapse analyses. Quantitative analysis of the local cell surface pH was performed between weakly alkaline and acidic pH (between pH 7.5 and 5.0). The probe detection range of FITC-PEG-lipid is suitable for monitoring pH in the various physiological and pathological events that occur usually at near neutral or weakly acidic pH. Especially at the pH around 6.0, where the fluorescence ratio changes almost linearly dependent on pH, the difference of 0.1 pH unit was able to be distinguished.

One of the advantages of FITC-PEG-lipid is in its remarkably simple experimental procedure for cell surface labeling, which is particularly beneficial for high-throughput screening *in vitro*. In the case of the genetically encoded probe^10^, the artificial gene encoding probe proteins has to be introduced into the cells. For the FRET probe utilizing the pH-dependent structural-switch of ssDNA^11^, amines on the plasma membrane have to be biotinylated prior to labelling because the probe is tethered at cell surface via biotin-streptavidin complex. To use the pH probe bound to pHLIP^13^ *in vitro*, cells need to be initially incubated with the probe at acidic pH, in order to insert pHLIP into the plasma membrane. In contrast, the cell surface can be labeled with FITC-PEG-lipid by a single step, i.e., by just adding it to the cell culture medium. The protocol is much simpler and has a less possibility of damaging cells compared with other methods. Regarding the simplicity of the labeling procedure, lipid–ssDNA-based probe^12^ is comparable to FITC-PEG-lipid. However, as pH-sensitive and -insensitive fluorophores are separately conjugated with lipid-ssDNA, two probe molecules have to be used in combination for ratiometry. FITC-PEG-lipid, in contrast, allows measurements with a single probe molecule due to the ratiometric fluorescent property of FITC. Using FITC-PEG-lipid, it would be possible to conduct a cell-based high-throughput screening, for example, of anticancer drugs targeting the extracellular acidity of cancer cells.

Concerning *in vivo* applications, the advantage of FITC-PEG-lipid is that the *in vivo* biocompatibility and stability of the PEG-lipid have been proved in transplantation experiments using animals^17,24–28^. In contrast, most of the above-mentioned pH probes, especially those employing DNA linkers^11,12^, need to be carefully assessed for their stability *in vivo*, considering the rapid degradation of DNA via the serum nucleases^29–32^. For the *in vivo* application of PEG–lipid-based probe, however, several important issues still remain to be addressed. First, for the efficient deep tissue imaging, fluorophores with near infrared excitation and emission are preferred in general. This is because of the high tissue transparency and the minimal autofluorescence at lower-energy wavelength^33^. Therefore, the replacement of FITC with near infrared fluorophores^34–36^ would be appropriate to promote the application of PEG–lipid-based probe for *in vivo* imaging. Second, FITC-PEG-lipid is non-selective and incorporated into any cells. Selective targeting of probes to the plasma membrane of particular cell types would be especially beneficial for clinical applications such as the cancer diagnosis, because non-selectively distributed probes would produce significant background signals. Recently, the acidic cell surface pH in mouse tumor model has been successfully measured by using a pHLIP-based probe^13^. To the best of our knowledge, this excellent study is the first *in vivo* cell surface pH imaging done by a ratiometric fluorescent probe. The key of the method was the selective delivery of the pH probe to the acidic tumor cell surface by pHLIP, a peptide selectively inserted into the plasma membrane at acidic pH. The applications *in vivo* would be accelerated if such a selective labeling can be conferred to PEG–lipid-based probe.

We also would like to emphasize that the potential of PEG–lipid-based probe is not limited to the analysis of pH. By conjugating PEG-lipid with appropriate indicator molecules, it would be possible to develop probes for the analysis of juxtamembrane local concentration and dynamics of various biologically important inorganic ions and organic small molecules. In addition, by modification of the labeling methods, PEG–lipid-based probes may be used for the investigation of the biological events at the intracellular and orgenellar juxtramembrane regions. For example, PEG–lipid-based probes can be applied intracellularly by using patch pipette in the whole-cell configuration of patch-clamp method, to label the inner leaflet of the plasma membrane and the surface of the organellar membranes.

In summary, our study has characterized FITC-PEG-lipid as a functional probe for the ratiometric fluorescent imaging of the cell surface pH. Due to its sensitive and reversible response, remarkably simple cell surface labeling procedure, and excellent spatiotemporal resolution, FTIC-PEG-lipid can be utilized to visualize and quantitatively monitor the local cell surface pH for various purposes. More generally, this study has verified that, in addition to its originally intended use for the cell surface modification in transplantation therapy, PEG-lipid is also applicable as the core structure for various bio-membrane-anchored probes designed for sensing the juxtamembrane environments.

## Methods

### Materials

FITC-PEG-lipid was synthesized from *α*-*N*-hydroxysuccinimidyl-*ω*-*tert*-butoxycarbonyl poly(ethylene glycol) (NHS-PEG-Boc, Mw: 5000 Da), 1,2-palmitoyl-*sn*-glycero-3-phosphatidylethanolamine (DPPE), and fluorescein isothiocyanate as described previously^16^. Fetal calf serum (FCS) was purchased from Gibco. Penicillin/streptomycin was purchased from NacalaiTesque. All the other reagents used for experiments were of the analytical grade from WAKO Chemicals unless otherwise specified. Plasmids, pcDNA3.1(+)-mem-mCherry and -mem-AcGFP, were constructed in this study. The encoded proteins, mem-mCherry and mem-AcGFP, consist of a fluorescent protein connected with an *N*-terminal peptide of mouse Lyn kinase (15 amino acids) via a Gly-Ser linker. The coding sequence was amplified from pmCherry-N1 and pAcGFP-N1 (Clontech) using the following common primer set: forward 5′-gcg*gaattc*gccaccATGGGATGTATTAAATCAAAAAGGAAAGACAATCTCAATGACGATggcagcatggtgagcaagggcgagga-3′ and reverse 5′-ggg*ctcgag*ttacttgtacagctcgtccatgc-3′, wherein the EcoRI restriction site in forward primer and the XhoI restriction site in reverse primer were indicated by italic letters. The sequence encoding the mouse Lyn *N*-terminal peptide was indicated by capital letters. The PCR product was digested with EcoRI and XhoI, and ligated with pcDNA3.1(+) (Invitrogen) linearized at EcoRI and XhoI sites using Ligation high ver.2 (TOYOBO).

### Cell culture

Human gastric signet-ring-cell carcinoma KATO III cells (Japanese Collection of Research Bioresources Cell Bank, Cell# JCRB0611) were cultured in RPMI-1640/E-MEM 1:1 medium supplemented with 10% FCS and penicillin/streptomycin at 37 °C with 5% CO_2_. The cells were transfected with the plasmid by using Lipofectamine 2000 (Invitrogen) one day after seeding and further cultured for 2 days. For the confocal laser scanning microscopy, cells were seeded on 35 mm glass bottom dish (Matsunami) coated with Cell-Tak Cell Tissue Adhesive (Corning).

### Spectrofluorometry

Fluorescence spectra of FITC-PEG-lipid were recorded in the solutions with various pH at room temperature using a spectrofluorometer F-7000 (Hitachi). FITC-PEG-lipid was suspended in HBSS [125 mM NaCl, 4.8 mM KCl, 1.2 mM MgSO_4_, 1.3 mM CaCl_2_, 5.6 mM glucose and 5 mM of buffer reagent (glycine/HCl for pH 2.0/3.0, acetic acid/sodium acetate for pH 4.0/4.5/5.0, MES/NaOH for pH 5.5/6.0/6.5, HEPES/NaOH for pH 7.0/7.5/8.0, and CAPS/NaOH for pH 9.0/10.0)] at the concentration of 1 μg/mL in a quartz cuvette. When indicated, NaCl and/or KCl were replaced with choline chloride. The excitation spectra at emission wavelength of 519 nm was recorded in the excitation scan (400–510 nm). For the emission scan (500–550 nm), the excitation wavelength was set at either 458 nm or 488 nm. The slit widths were 5 nm for both excitation and emission. PMT voltage was set at 950 V and scan speed was 240 nm/min.

### Confocal laser scanning microscopy

Cells were washed twice in HBSS (pH 7.0) and incubated with FITC-PEG-lipid (0.1 mg/mL in HBSS, pH 7.0) at room temperature for the indicated period. After washing twice in HBSS (pH 7.0), the dishes were subjected to confocal laser scanning microscopy (LSM710, Zeiss). For the time course of the cell surface labeling, image acquisition settings were as follows: BP 500–550 nm filter, 2% Argon 488 nm laser power, C-Apochromat 63×/1.20 W Corr M27 objective lens, 0.79 μsec pixel^−1^ dwell, 4096 × 4096 pixel resolution, ×1 zoom (pixel size: 0.033 × 0.033 μm), 16-bit intensity resolution, 90 μm pinhole diameter, average of 4 scans per image. To compare the localization of FITC-PEG-lipid and mem-mCherry, images were acquired with the following settings: BP 500–550 nm filter for FITC and BP 575–610 filter for mCherry, 2% Argon 488 nm laser power for FITC and 2% DPSS 561 nm laser power for mCherry, C-Apochromat 63×/1.20 W Corr M27 objective lens, 0.79 μsec pixel^−1^ dwell, 4096 × 4096 pixel resolution, ×2 zoom (pixel size: 0.016 × 0.016 μm), 16-bit intensity resolution, 90 μm pinhole diameter, average of 4 scans per image. The fluorescence peaks across the plasma membrane was detected by using Zen 2012 software (Zeiss).

For the static analyses of the fluorescence ratio, images were acquired with the following settings: BP 500–550 nm filter, 3% Argon 458 nm and 1% Argon 488 nm laser power, Plan-Apochromat 20×/0.8 M27 objective lens, 0.79 μsec pixel^−1^ dwell, 2048 × 2048 pixel resolution, ×2 zoom (pixel size: 0.104 × 0.104 μm), 16-bit intensity resolution, 90 μm pinhole diameter, average of 4 scans per image. To quantify the local extracellular pH, drift correction was performed using ImageJ (NIH, http://rsb.info.nih.gov/ij/) with Template Matching and Slice Alignment plugin (https://sites.google.com/site/qingzongtseng/template-matching-ij-plugin). The fluorescence intensities in ROIs set on the plasma membrane were calculated by ImageJ, after the subtraction of background fluorescence intensities in the regions where no cells exist. For time-lapse analysis, the external solution was changed by a stage-top perfusion system. Images were acquired every 10 sec for 380 sec (39 frames) with the following settings: 512 × 512 pixel resolution, ×8 zoom (pixel size: 0.104 × 0.104 μm), average of 2 scans per image. The other settings are same as for the static ratiometry. Pseudocolored ratiometric images were generated by using Zen 2012 software (Zeiss).

### Regression analysis

Curve fitting by non-linear regression analyses for all the graphs was performed by SigmaPlot 13 (Systat Software).

### Data availability

All data generated or analysed during this study are available from the corresponding author on reasonable request.

## Electronic supplementary material

Supplementary information

Supplementary Video S1
