# Supplementary material for: Ratiometric fluorescence imaging of cell surface pH by poly(ethylene glycol)-phospholipid conjugated with fluorescein isothiocyanate
Source: Sci Rep. 2017 Dec 13;7:17484. doi: 10.1038/s41598-017-17459-y (PMC5727509; doi:10.1038/s41598-017-17459-y)
Supplement: Supplementary file 1 — Supplementary information [file 41598_2017_17459_MOESM1_ESM.pdf]

Supplementary information

Ratiometric fluorescence imaging of cell surface pH by poly(ethylene glycol)-phospholipid conjugated with fluorescein isothiocyanate

Ryuichi Ohgaki[†], *Yuji Teramura*[‡,//], *Daichi Hayashi*[†], *Lili Quan*[†], *Suguru Okuda*[†], *Shushi Nagamori*[†], *Madoka Takai*[‡] *and Yoshikatsu Kanai*[*,†]

[†] Department of Bio-system Pharmacology, Graduate School of Medicine, Osaka University, 2-2 Yamadaoka, Suita, Osaka 565-0871, Japan.

[‡] Department of Bioengineering, The University of Tokyo, 7-3-1 Hongo, Bunkyo-ku, Tokyo 113-8656, Japan.

[//] Department of Immunology, Genetics and Pathology (IGP), Rudbeck Laboratory C5:3, Uppsala University, SE-751 85 Uppsala, Sweden.

Supplementary Figure

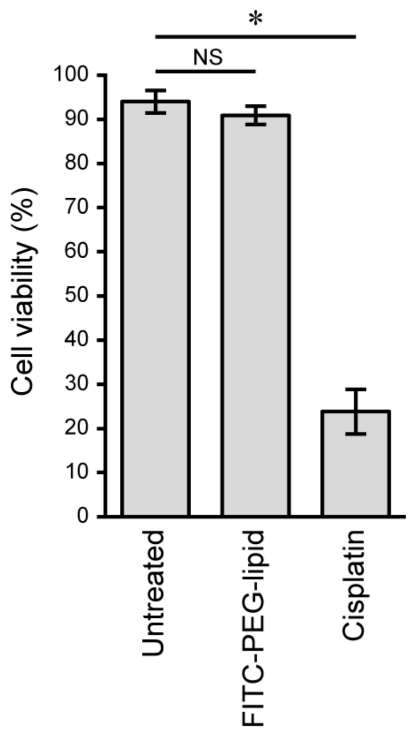

Supplementary Figure S1. Effect of FITC-PEG-lipid on cell viability. KATOIII cells incubated with FITC-PEG-lipid and untreated cells were subjected to trypan blue exclusion assay. Viability of cisplatin-treated cells were analyzed as a control. Data shown are mean ± SD (n = 4). Statistical analysis was performed by one-way ANOVA followed by Bonferroni post-hoc tests for comparison with untreated cells. * p <0.05. NS, not significant.

Supplementary Video Legends

Supplementary Video S1. Time-lapse ratiometric fluorescence analysis of the extracellular pH. Movie file was constructed from the pseudocolored images shown in Fig. 5 using ImageJ software. Interval: 10 sec. Frame rate: 3 fps.

Supplementary Methods

Cell viability analysis by trypan blue exclusion assay

KATOIII cells were washed twice and incubated in HBSS (pH 7.0) with or without FITC-PEG-lipid (0.1 mg/mL) at room temperature for 30 min. After washing twice in HBSS (pH 7.0), cell were seeded in 12-well plate at the density of 4×10^4 cells/well and cultured for 72 hours. Cells were collected and mixed with 0.4% trypan blue solution at 1:1 volume ratio. The viability was calculated as the percentage of trypan blue-negative cells. As a control of cytotoxic compound, cisplatin was added to the medium at 20 μg/mL.
